# Meta-Analysis of Organic Fertilization Effects on Soil Bacterial Diversity and Community Composition in Agroecosystems

**DOI:** 10.3390/plants12223801

**Published:** 2023-11-08

**Authors:** Xiangyang Shu, Weijia Liu, Han Huang, Qinxin Ye, Shunxi Zhu, Zhaohui Peng, Yiding Li, Liangji Deng, Zepeng Yang, Honglin Chen, Dinghui Liu, Jialing Shi

**Affiliations:** 1 Key Lab of Land Resources Evaluation and Monitoring in Southwest, Ministry of Education, Sichuan Normal University, Chengdu 610068, China; 2 Institute of Agricultural Bioenvironment and Energy, Chengdu Academy of Agriculture and Forestry Sciences, Chengdu 611130, China; liuweijia27@163.com (W.L.); yeqinxin123@163.com (Q.Y.); zsx25x29@163.com (S.Z.); 3 College of Economics and Management, Xinjiang Agricultural University, Urumqi 830052, China; 18328717493@163.com; 4 College of Resources, Sichuan Agricultural University, Chengdu 611130, China; encili@foxmail.com (Y.L.); auh6@sicau.edu.cn (L.D.); 5 Soil and Fertilizer Research Institute, Sichuan Academy of Agricultural Sciences, Chengdu 610066, China; zepengyang@126.com (Z.Y.); chenhl0107@163.com (H.C.); dinghuiliu@163.com (D.L.); 6 Chengdu Agricultural and Rural Bureau, Chengdu 610066, China; 19938264525@163.com

**Keywords:** organic fertilization, bacterial diversity, soil acidity, copiotrophic bacteria, agroecosystems

## Abstract

Application of organic fertilizers or their combination with chemical fertilizers is a feasible practice for improving soil fertility and reducing soil degradation in agroecosystems, and these regulations are mainly mediated though soil microbial communities. Despite bacteria ranking among the most abundant and diverse groups of soil microorganisms, the effects of long-term organic fertilization (OF) and chemical–organic fertilization (COF) on soil bacterial diversity and community composition remain unclear. In this study, we conducted a meta-analysis and demonstrated that OF had no significant effect on bacterial alpha diversity. Application of chemical fertilizer and crop residue significantly decreased bacterial Richness index. Both OF and COF significantly altered bacterial community structure, with these changes being predominately attributed to shifts in soil pH. For bacterial phyla, both OF and COF significantly increased the relative abundance of *Proteobacteria* and *Bacteroidetes*, suggesting that OF and COF may cause the enrichment of copiotrophic taxa. In addition, COF significantly increased the relative abundance of *Gammaproteobacteria* but decreased the relative abundance of *Acidobacteria*. Overall, our results suggest that organic and chemical–organic fertilization can effectively maintain bacterial diversity and enhance soil fertility in agroecosystems, and the alteration of soil bacterial community structure is closely intertwined with soil pH.

## 1. Introduction

Optimal agricultural management practices are essential to improving soil fertility and sustaining crop productivity in agricultural ecosystems [1]. Over the past few decades, high-intensity agricultural practices, such as the long-term excessive application of chemical nitrogen (N) fertilizers, have resulted in a noticeable decline in soil quality and microbial diversity, which is a major issue for agricultural productivity and sustainability [2,3]. Therefore, the development of low-input, high-productivity agricultural nutrient management practices has become increasingly important. Organic materials (e.g., animal manure, crop residue, compost, or their combinations) (OF) or chemical-plus-organic fertilization (COF) have been proposed as alternatives to chemical fertilization due to its perceived advantages in sustainability and environmentally friendly properties [4]. Previous studies have demonstrated that the application of organic or chemical–organic fertilizers in agricultural soil can lead to benefits such as increasing soil carbon sequestration and fertility, and improving microbial functions [5,6,7]. However, the impacts of organic and chemical–organic fertilizer application on soil microbial diversity remain largely elusive.

Soil bacteria are likely the most abundant and diverse community on our planet [8,9]. Bacterial diversity (that is, taxonomic diversity, Richness, and community composition) plays a critical role in mediating a range of ecosystem processes, including organic matter decomposition, nutrients biogeochemical cycling, climate regulation, and plant growth [10,11,12,13]. Therefore, maintaining a high level of bacterial diversity in soil is essential for the health and sustainable development of agriculture. However, it is also a challenging task because soil bacterial communities are changing at an unprecedented rate due to environmental changes caused by common agricultural management practices, particularly long-term nutrient addition [14,15,16,17].

Long-term fertilization can alter soil physicochemical properties, crop productivity, and microbial activities and interactions [18,19,20]. To date, the impact of fertilization on soil microbial activities and fungal diversity has been documented [21,22]. However, studies focusing on the response of bacterial diversity and community structure remain fragmented and require a comprehensive synthesis. For instance, organic or chemical–organic fertilization may or may not increase soil bacterial diversity [23,24,25]. Long-term fertilization has also been found to change bacterial community structure and is considered to be mainly due to changes in soil pH or soil carbon and/or nutrient availability caused by fertilization [26]. Furthermore, the effect of fertilization on the relative abundance of some key bacterial phyla remains controversial and highly variable under different land use types, fertilizer regimes, and experiment conditions [27,28,29]. The large heterogeneity of these results limits our understanding of the general bacterial diversity and community structure responses to organic and chemical–organic fertilization.

Here, we performed a meta-analysis based on 86 published papers with 183 observations to evaluate the effects of organic and chemical–organic fertilization on bacterial diversity, community structure and specific bacterial phyla. The objectives of our study were to answer the following questions: (1) How does organic and chemical–organic fertilization affect soil bacterial diversity and community structure? (2) How does organic and chemical–organic fertilization affect specific bacterial phyla? (3) What factors influence bacterial diversity, community structure, and specific bacterial phyla in response to organic and chemical–organic fertilization? We hypothesize that long-term organic and chemical–organic fertilization can maintain bacterial diversity and shift community structure. In addition, organic and chemical–organic fertilization may cause the enrichment of copiotrophic bacteria through increasing C and nutrient availability.

## 2. Results

### 2.1. Response of Soil Properties to Different Fertilizer Regimes

**Figure 1:**
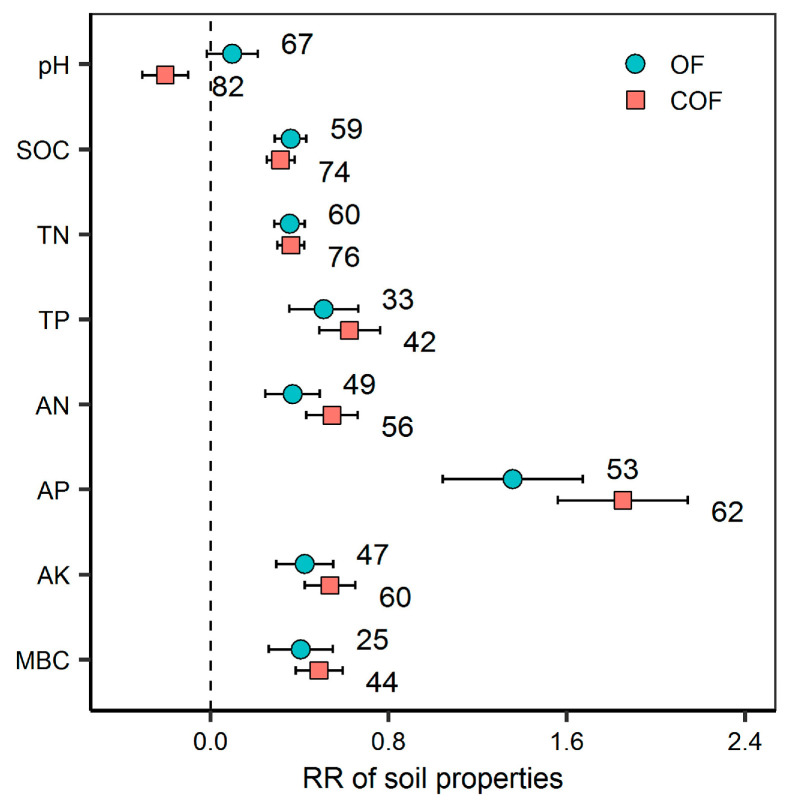
Effect of organic and chemical–organic fertilization on soil properties. Values are mean 95% confidence intervals of the percentage effects between the treatment and control. The numbers at the right side of the confidence intervals represent the sample sizes. SOC, TN, TP, AN, AP, AK and MBC represent soil organic carbon, total nitrogen, total phosphorus, available nitrogen, available phosphorus, available potassium and microbial biomass carbon, respectively. OF and COF represent organic fertilizer and chemical–organic fertilizer, respectively.

Compared to no fertilizer control, both OF and COF significantly increased SOC (43.1% for OF and 36.7% for COF), TN (43.2% and 42.4%), TP (86.5% and 66.0%), AN (72.3% and 44.3%), AP (536.2% and 288.1%), AK (70.8% and 52.4%), and MBC (62.6% and 49.7%) content (*p* < 0.05) (Figure 1). COF significantly decreased soil pH (*p* < 0.05). Fertilizer type was an important factor that affecting the response of soil pH, SOC, TN, TP, and AP to fertilization (*p* < 0.05). Manure application significantly increased soil pH by 0.18, but NPK + crop residue and NPK + manure application significantly decreased soil pH on average by −0.33 and −0.19, respectively (*p* < 0.05) (Supplementary Figure S1).

### 2.2. Response of Bacterial Diversity and Community Structure to Different Fertilizer Regimes

**Figure 2:**
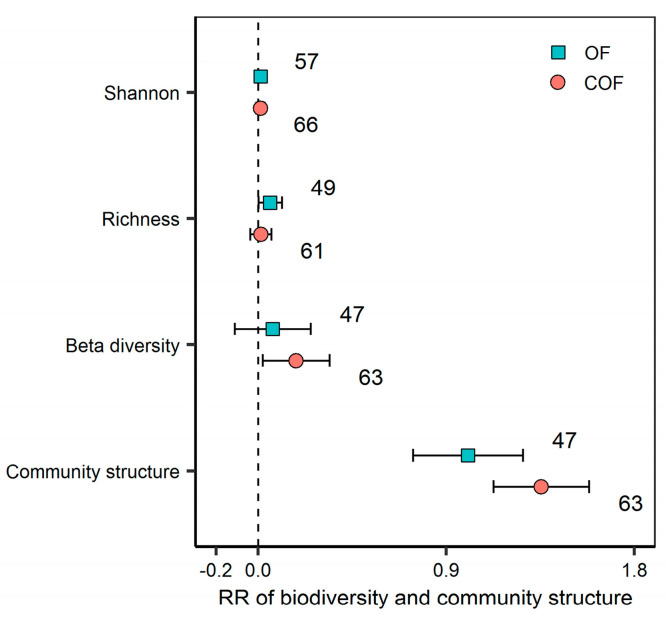
Effect of organic and chemical–organic fertilization on bacterial diversity and community structure. Values are mean ± 95% confidence intervals of the percentage effects between the treatment and control. The numbers at the right side of the confidence intervals represent the sample sizes.

**Figure 3:**
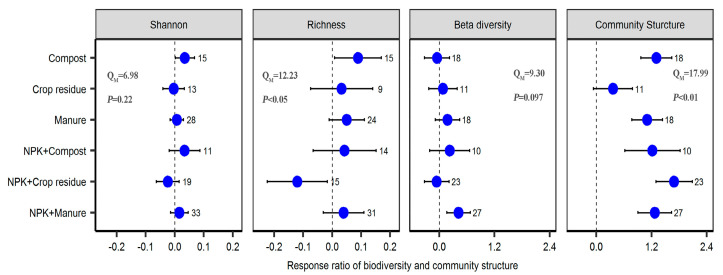
Effect of different fertilizer types on bacterial diversity and community structure. Values are mean ± 95% confidence intervals of the percentage effects between the treatment and control. The numbers at the right side of the confidence intervals represent the sample sizes.

**Figure 4:**
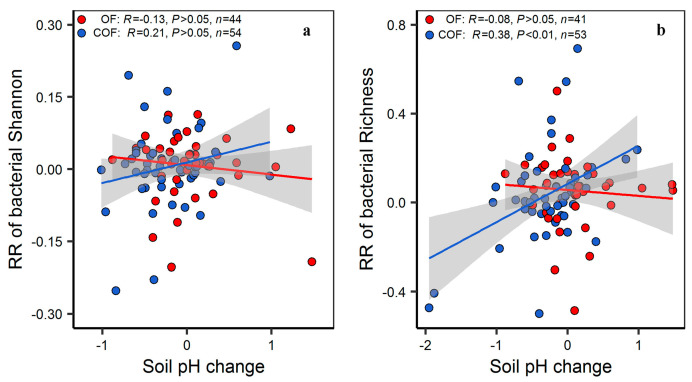
Coordinated changes between soil pH and bacterial diversity. (**a**) Relationship between changes in soil pH and response ratio of bacterial Shannon. (**b**) Relationship between changes in soil pH and response ratio of bacterial Richness.

Both OF and COF significantly changed bacterial community structure, but had no significant effect on the bacterial Shannon and Richness index. COF significantly increased bacterial beta-diversity (*p* < 0.05) (Figure 2). Fertilizer types had a significant effect on the magnitude of bacterial Richness and community structure response. Compost application significantly increased bacterial Richness (on average by 9.3%), whereas NPK + crop residue application significantly decreased bacterial Richness (−11.3%) (*p* < 0.05) (Figure 3). The response of bacterial community structure to both OF and COF was significantly varied with land use types (*p* < 0.05) (Supplementary Figure S2). Response ratios of bacterial Richness were positively correlated with soil pH changes under COF (*p* < 0.01) (Figure 4). Response ratios of bacterial Shannon were positively correlated with bacterial Richness under both OF and COF (*p* < 0.001) (Supplementary Figure S3).

### 2.3. Response of Specific Bacterial Phyla to Different Fertilizer Regimes

**Figure 5:**
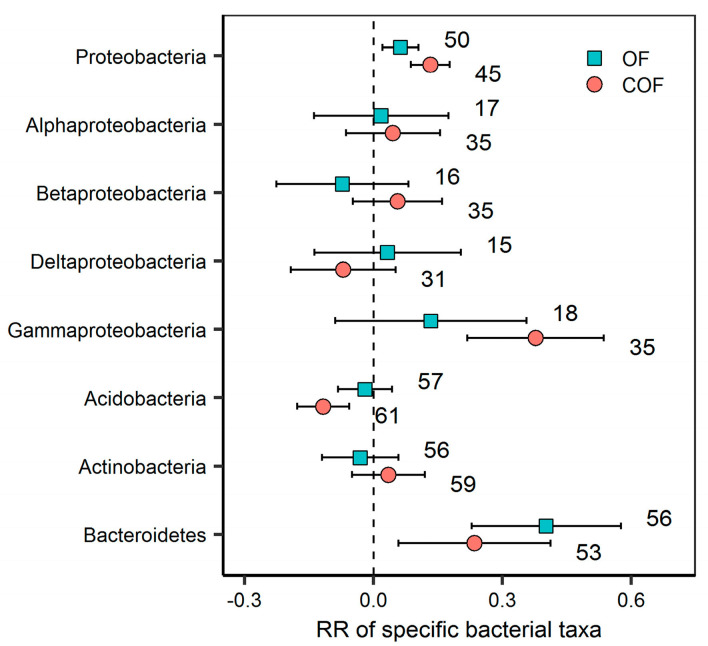
Effect of organic and chemical–organic fertilization on specific bacterial phyla. Values are mean ± 95% confidence intervals of the percentage effects between the treatment and control. The numbers at the right side of the confidence intervals represent the sample sizes.

**Figure 6:**
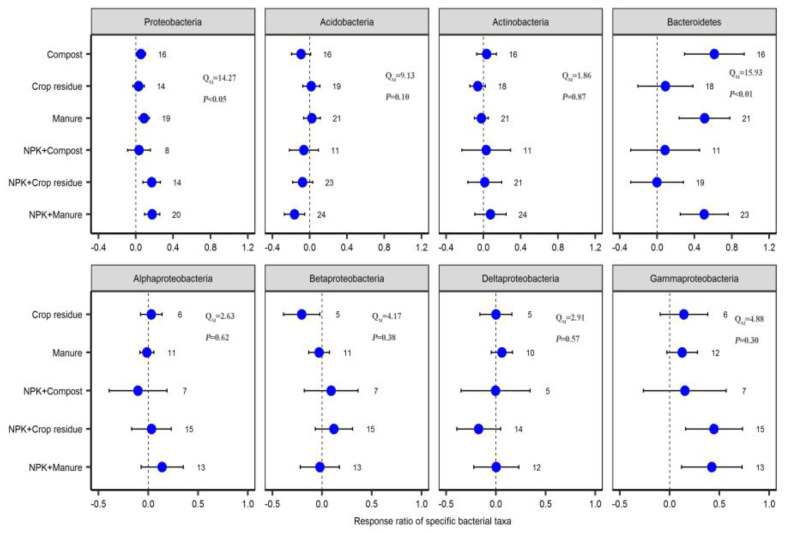
Effect of different fertilizer types on specific bacterial phyla. Values are mean ± 95% confidence intervals of the percentage effects between the treatment and control. The numbers at the right side of the confidence intervals represent the sample sizes.

Both OF and COF significantly increased the relative abundance of *Proteobacteria* (6.5% for OF and 14.1% for COF) and *Bacteroidetes* (49.4% and 26.5%) (*p* < 0.05). COF significantly increased the relative abundance of *Gammaproteobacteria* (45.8%), but significantly decreased the relative abundance of *Acidobacteria* (11.1%) (*p* < 0.05) (Figure 5). Fertilizer type was an important factor in affecting the response ratio of *Proteobacteria* and *Bacteroidetes* to OF and COF (*p* < 0.05) (Figure 6). The responses of *Proteobacteria*, *Betaproteobacteria*, and *Gammaproteobacteria* to COF significantly varied with land use types (*p* < 0.05) (Supplementary Figure S4). Response ratios of *Bacteroidetes* were positively correlated with soil pH, SOC, and AP changes under OF and COF (*p* < 0.05). Response ratios of *Proteobacteria* were positively correlated with soil pH and TN changes under OF (*p* < 0.05). Response ratios of *Proteobacteria* were positively correlated with soil pH and AP changes under COF (*p* < 0.05) (Supplementary Figure S5).

### 2.4. The Importance of the Predictors of Different Fertilizer Regimes Effect on Bacterial Diversity and Community Structure

**Figure 7:**
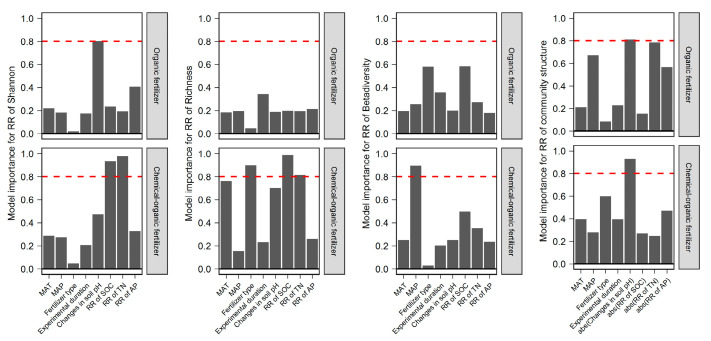
Model-averaged importance of the predictors of organic and chemical–organic fertilization impact on soil bacterial community. The importance is based on the sum of Akaike weights derived from the model selection using Akaike’s Information Criteria corrected for small samples. The red dotted line was a cutoff of 0.8. A cutoff of 0.8 is set to differentiate between important and non-essential predictors. Abs represents absolute value.

The changes in SOC and TN by COF were important predictors for the response ratio of bacterial Shannon and Richness. The absolute values of changed soil pH by OF and COF was the most important predictor for the changes in bacterial community structure. Besides, the changes in soil pH by OF was most important predictor for the response ratio of bacterial Shannon (Figure 7, Supplementary Figures S7 and S8). Response ratios of *Gammaproteobacteria* were positively correlated with SOC, TN, TP, and AP changes under COF (*p* < 0.05) (Supplementary Figure S9).

## 3. Discussion

### 3.1. Response of Specific Bacterial Diversity and Community Structure to Organic and Chemical–Organic Fertilization

Microbial diversity is a significant determinant of ecosystem health, as soil microorganisms play essential roles in maintaining ecosystem stability and productivity [30,31]. Despite the importance of bacterial diversity for soil fertility and plant growth, the long-term effects of organic and chemical–organic fertilization on bacterial diversity remain unclear, e.g., positive effects [23,32] or no/negative effects [19,33]. Our meta-analysis demonstrates that, compared to the no-fertilization control, OF and COF significantly altered bacterial community structure but had a negligible positive effect on the bacterial Shannon and Richness indices. Additionally, only COF showed a significant increase in increase bacterial beta diversity. These results imply that OF or COF had the potential to maintain soil bacterial diversity in agroecosystems.

At present, fertilization-induced variation of edaphic variables (e.g., pH, SOC and nutrient concentration, and moisture) associated with changes in bacterial diversity and community structure remains contradictory [26]. Model selection analysis revealed that the changes in soil pH were the most predominant predictor for bacterial Shannon in response to OF. When organic fertilizer combined with chemical fertilizer application, the changed SOC and TN were the important factors determining bacterial Shannon. Surprisingly, we observed that negative effect of SOC and positive of soil TN on bacterial alpha diversity under chemical–organic fertilization (Supplementary Figure S6). The negative effect of SOC on bacterial alpha diversity may be attributed to the high availability of a rich-C substrate under COF, triggering the competitive interaction with copiotrophic and oligotrophic bacteria in the bacterial communities. This competitive process may exclude some species from the bacterial community—resulting in a loss of bacterial diversity [34,35]. Soil pH changed by OF and COF was the most predominant predictor for bacterial community structure, which is consistent with many previous studies at local sites and global scales [11,36,37]. The explanation is that pH plays an important role in protein stability and pH homeostasis of cells and thus directly imposes a physiological constraint on soil bacterial communities. This constraint decreases the net growth of some bacterial taxa, which cannot survive when the soil pH falls outside a particular niche and thus change the results of the competition [16].

Fertilizer type plays a crucial role in influencing the activities of the soil-dwelling microorganisms [38]. A recent meta-analysis has highlighted the significant effect of the type of chemical fertilizers on the magnitude and direction of microbial diversity and community structure response [16]. In this study, our meta-analysis indicated that the influences of OF and COF on bacterial Richness and community structure vary significantly depending on the fertilizer types used. One possible explanation for these observations is the considerable heterogeneity in fertilizer properties and application rates [6,39]. These differences in fertilizer application may create unique microenvironment for soil microorganisms, leading to alterations in microbial diversity and community structure. For example, application of compost and manure alongside crop residue can mitigate soil acidification, and increases SOC and nutrient concentration. Notably, the positive relationship between the response ratios of bacterial Richness and soil pH, suggesting that long-term NPK + crop residue application may not be a feasible practice that can stably maintain bacterial Richness in global agroecosystems (Supplementary Figure S8).

### 3.2. Response of Specific Bacterial Phyla to Organic and Chemical–Organic Fertilization

Species composition, an important indicator of the bacterial community, is crucial for maintaining ecosystem functioning [12,40]. Previous studies have suggested that changes in soil physicochemical characteristics and substrate utilization resulting from fertilization are unlikely to uniformly impact the species composition of bacterial and fungal communities equally [14,22]. Our findings revealed that both OF and COF significantly increased the relative abundance of *Proteobacteria* and *Bacteroidetes*. Generally, *Bacteroidetes* and many of the dominant *Proteobacteria* are classified as copiotrophic taxa characterized by a fast growth rate and r-selected life strategy. These taxa thrive in conditions with elevated carbon availability and prefer to utilize more labile C substrates [41,42]. Hence, the enhancement of soil C and nutrient availability under OF and COF may stimulate the growth of these copiotrophic bacteria. Additionally, the significantly positive relationship observed between *Proteobacteria* and *Bacteroidetes* and soil pH under OF and COF suggests that the relative stable soil pH in such conditions may provide an ideal environment for the proliferation of these copiotrophic bacteria. Upon examining *Proteobacteria* at the class level of taxonomic resolution, we noted various responses among four classes to fertilizer addition. Specifically, only COF significantly increased the relative abundance of *Gammaproteobacteria*. This observation aligns with the tendency of *Gammaproteobacteria* to be copiotrophic taxa [42,43]. The positive correlation of *Gammaproteobacteria* with SOC, TN, and TP under COF further suggests that the increased availability of soil C and nutrients likely contributed to the higher relative abundance of *Gammaproteobacteria* (Supplementary Figure S9). We propose that further investigation is needed to explore the effects of OF and COF on bacterial community composition at more specific phylogenetic resolution levels.

*Acidobacteria* are typically classified as oligotrophic taxa characterized by a slower growth rate and K-selected life strategy, which are taxa that prefer soil environments with low carbon availability and may have the ability to metabolize nutrient-poor and recalcitrant C substrates [41,42,44]. Our results showed that COF significantly decreased the relative abundance of *Acidobacteria*, whereas this effect was not observed with OF. The significant negative correlation observed between of the RR of *Acidobacteria* and AP under COF suggests that an increase in AP may lead to a decrease in the relative abundance of *Acidobacteria*. This result is consistent with previous studies [45,46], which have found that phosphorus fertilization can decrease the relative abundance of *Acidobacteria*. Furthermore, the application of N fertilizer may hinder the metabolic process of *Acidobacteria* for C substrates that consequence inhibited the growth of *Acidobacteria* [43].

The RR of *Proteobacteria* and *Bacteroidetes* significantly varied with fertilizer types. The RR of *Proteobacteria* under COF was higher than that of OF. One possible explanation for this observation is that compared to OF, COF can more effectively improve soil microbial C use efficiency and nutrient availability [20,47]. Moreover, we found a higher increase in *Bacteroidetes* abundance under the application of compost, manure, and their combination with NPK fertilizer than under crop residue and NPK + crop residue application. Previous studies indicated that *Bacteroidetes* are important and dominant carbohydrate degraders in soil environments [9]. At the same time, compost manure and manure had relatively higher carbohydrate and nutrient contents than crop residue [6]. Therefore, the *Bacteroidetes* thrive under compost and manure application because of their ability to secrete diverse arrays of carbohydrate-active enzymes which target the compost or manure-derived carbohydrate in soils. In addition, the RR of *Bacteroidetes* under both OF and COF had a significant positive correlation with the application rate of organic fertilizer, suggesting that the increase in *Bacteroidetes* had a closer correlation to organic amendment, compared to other phyla (Supplementary Table S1). Apart from fertilizer types, land use types can alter the response of *Proteobacteria*, *Betaproteobacteria*, and *Gammaproteobacteria* to chemical–organic fertilization. We found a higher relative abundance of *Proteobacteria*, *Betaproteobacteria*, and *Gammaproteobacteria* abundance in upland fields than in paddy fields. This result can be attributed to the fact that upland fields have better aeration conditions, which are beneficial to the decomposition of organic materials, and then the release of nutrients consequently stimulates the growth of copiotrophic taxa [48].

## 4. Materials and Methods

### 4.1. Data Collection

An extensive literature survey was conducted through Scopus (Elsevier), Web of Science (Thomson Reuters), Google Scholar (scholar.google.com) and China National Knowledge Infrastructure databases (www.cnki.net) until March 2022 with no restriction on publication year. We performed a literature search using terms (fertiliz* OR organic amendment OR straw return OR manure OR compost) AND (bacteria*). The following criteria were used to select studies: (1) only field studies of organic or chemical–organic fertilization are selected; (2) at least one bacterial variable, including diversity (i.e., OTU (operational taxonomic units) Richness, Chao 1 and/or Shannon–Wiener index), community structure and/or specific phyla reported; (3) the duration of experiments is ≥2 years; (4) if one paper reported various independent experiments, each of them was considered as an individual study and incorporated as an independent observation into our dataset; (5) if one paper contained results from various sampling dates and soil depths, we only used the data from the latest sampling time-point and from the sample collected from the uppermost layer of soil; (6) besides the bacterial variables, soil properties including soil pH, soil organic carbon (SOC), total nitrogen (TN), total phosphorus (TP), available nitrogen (AN), available phosphorus (AP), available potassium (AK), and microbial biomass carbon (MBC) were also extracted if they appeared in studies selected in accordance with the above papers.

The location (latitude and longitude), climate factor (mean annual temperature (MAT) and mean annual precipitation (MAP)), edaphic parameters (soil pH, soil organic carbon, soil total nitrogen, clay content), land use type, fertilizer type, application rate of organic, N, P and K fertilizer, and experimental duration were collected for each site. pH-CaCl_2_ was used instead of pH-H_2_O in the paper [49]. If available nitrogen was not reported, we use the sum of ammonium nitrogen and nitrate nitrogen instead. Missing mean annual temperature (MAT) and mean annual precipitation (MAP) were extracted from the database at http://epsweb.larc.nasa.gov (accessed on 15 October 2023) using the site information. Overall, we collected 183 paired observations of organic fertilizer and synthetic fertilizer from 86 studies (Supplementary Materials dataset1).

Land use types were classified into two categories: paddy and upland. Types of fertilizer included compost, crop residue, manure, crop residue plus manure, and their combination with NPK fertilizer. The experimental duration was the range from 2 to 110 years. The application rate of organic fertilizer was the range from 0.4 to 100 Mg ha^−1^, respectively. Initial soil pH, organic carbon, total nitrogen, and clay content were the range from 4.5 to 8.7, 2.5 to 24.0 g kg^−1^, 0.14 to 2.01 g kg^−1^, and 2.2 to 70.0%, respectively.

### 4.2. Meta-Analysis

The natural log of response ratio (*RR*) was used to evaluate the responses of soil bacterial α-diversity (i.e., Shannon and Richness), specific phyla, and soil properties to organic and chemical–organic fertilization. The *RR* was calculated according to the following equation:

*RR* = ln X_t_ − ln X_c_ (1)

where X_t_ and X_c_ denote the average values of selected variables in the fertilizer treatment and no fertilizer control, respectively. It is worth noting that the response of soil pH to organic and chemical–organic fertilization was represented as a change in soil pH [16].

The natural log of response ratio (*RR*) of bacterial β-diversity and community structure was calculated according to the following equation:

(2) $${\mathrm{RR}}_{\mathrm{Beta}}=\ln\;{\bar{D}}_{t}-\ln\;{\bar{D}}_{c}$$

(3) $${\mathrm{RR}}_{\mathrm{structure}}=ln\left(\frac{\bar{D_{b}}}{\bar{D_{t}+D_{c}}}\right)$$

where $\bar{D}$*_t_*, $\bar{D}$*_c_*, $\bar{D}$*_b_* and $\bar{D_{t}+D_{c}}$ denote the average values of the Euclidean distances within fertilizer treatment (*D_t_*), Euclidean distances within no fertilizer control (*D_c_*), and Euclidean distances between no fertilizer and fertilizer treatment (*D_b_*), respectively.

The observations’ variances (ν) were calculated according to the following equation:

(4) $$\nu =\frac{S_{t}^{2}}{N_{t}X_{t}^{2}}+\frac{S_{c}^{2}}{N_{c}X_{c}^{2}}$$

where *N_t_* and *Nc* denote the sample size of the variable in the fertilizer treatment and no fertilizer treatment, respectively; *S_t_* and *S_c_* denote the standard deviations (SD) of the variable in the fertilization and no fertilizer control, respectively. Missing standard deviations were calculated using the average coefficient of variation of the datasets with known standard deviations [50].

In our meta-analysis, we used the random-effect model to calculate the overall natural the log of response ratio and corresponding 95% confidence intervals of variables to different fertilizer regime with a moderator of fertilizers application. Furthermore, we use the random-effect model to compare the natural log of response ratio of variables to each fertilizer regime among land use types and fertilizer types using the omnibus test (Q_M_). In subgroup analysis, groups with less than 5 sample sizes will be removed. Since converting continuous variables into discrete groups will reduce statistical power, we used linear regression to analyze the relationship between different climatic factors (MAT and MAP), initial soil properties (pH, SOC, TN and clay content), experimental conditions (including experimental duration and organic fertilizer rate) and bacterial variables under organic and chemical–organic fertilization (Supplementary Tables S1 and S2).

### 4.3. Model Selection Analysis

Model selection was based on Akaike information criterion (AIC) corrected [16]. Three types of candidate predictors were included in our analysis: (1) experimental conditions, including fertilizer type and experimental duration; (2) climate factors, including mean annual temperature and mean annual precipitation; and (3) changes in edaphic variables, including changes in soil pH, the log of response ratio of soil organic carbon, total nitrogen, available nitrogen and available phosphorus. For bacterial community structure, the absolute values of changes in soil pH and the log of response ratio of soil organic carbon, total nitrogen, available nitrogen, and available phosphorus were used in the model selection analysis. All data analysis was performed in R 4.0.2 (R Core Team, 2020).

## 5. Conclusions

In summary, our meta-analysis demonstrates that long-term organic and chemical–organic fertilization can improve soil fertility, maintain soil bacterial biodiversity, and alter bacterial community structure in agroecosystems. Both organic and chemical–organic fertilization favor the growth of copiotrophic bacteria. Land use types and fertilizer types contributed to the variabilities of response ratio of bacterial community structure, and organic and chemical–organic fertilization shaped bacterial community structure predominately by changing the soil pH. Overall, adopting long-term organic or chemical–organic fertilization can consistently maintain soil bacterial diversity and improve soil fertility. This highlights the potential of organic or chemical–organic fertilization as a sustainable fertilizer management practice in crop production. Further research should aim to (1) reveal the response of bacterial community composition to organic and chemical–organic fertilization at finer phylogenetic resolution levels and (2) clarify the response of bacterial functional diversity and bacterial species interaction to different fertilizer management practices.

## Data Availability

Original data may be provided upon request.

## Supplementary Materials

The following supporting information can be downloaded at https://www.mdpi.com/article/10.3390/plants12223801/s1. Figure S1: Effect of different fertilizer types on soil properties; Figure S2: Response of bacterial community structure to different land use types; Figure S3: Relationship between changes in soil properties and response ratio of bacterial diversity and community structure; Figure S4: Response of specific bacterial taxa to different land use types; Figure S5: Relationship between changes in soil properties and response ratio of specific bacterial phyla; Figure S6: Weighted averages of the model coefficients across various models for the responses of bacterial community to organic and chemical compound fertilization; Figure S7: Weighted averages of the model coefficients across various models for the responses of bacterial community to organic fertilization; Figure S8: Relationship between changes in soil properties and response ratio of bacterial Richness under NPK + crop residue application; Figure S9: Relationship between changes in soil properties and response ratio of Proteobacterial subclass; Table S1: Correlation coefficients between response of specific bacterial taxa and climate factors, soil properties, and fertilizer regimes; Table S2: Correlation coefficients between response of bacterial alpha diversity and climate factors, soil properties, and fertilizer regimes. Dataset1: The original data of meta-analysis.
