# Supplementary material for: Meta-Analysis of Organic Fertilization Effects on Soil Bacterial Diversity and Community Composition in Agroecosystems
Source: Plants (Basel). 2023 Nov 8;12(22):3801. doi: 10.3390/plants12223801 (PMC10675672; doi:10.3390/plants12223801)
Supplement: Supplementary file 1 [file plants-12-03801-s001.zip › Supplementary materials.pdf]

Supplementary Table

Supplementary Table 1 Correlation coefficients between response of specific bacterial taxa and climate factors, soil properties, and fertilizer regimes.

Fertilizer regime	Factor	RR of *Proteobacteria*	RR of *Acidobacteria*	RR of *Actinobacteria*	RR of *Bacteroidetes*
Organic fertilizer	MAT	0.04(50)	-0.1(57)	-0.24(56)	0.27(56)*
	MAP	0.22(50)	-0.15(57)	-0.53(56)***	0.43(56)***
	Soil pH	-0.26(22)	-0.12(26)	-0.06(26)	-0.33(26)
	SOC	-0.29(22)	0.17(26)	-0.16(26)	-0.42(26)*
	TN	-0.05(21)	0.17(25)	-0.23(25)	-0.21(25)
	Clay content	-0.47(7)	0.14(9)	-0.38(8)	-0.85(8)**
	Experimental duration	0.04(50)	0.09(57)	0.2(56)	-0.08(56)
	organic fertilizer rate	0.17(46)	0.12(49)	0.14(48)	0.35(48)*
Chemical-organic fertilizer	MAT	-0.01(45)	0.16(61)	0.27(59)*	0.06(53)
	MAP	0.1(45)	0.1(61)	0.32(59)*	0.17(53)
	Soil pH	0.01(24)	0.37(27)	-0.22(27)	-0.58(23)**
	SOC	-0.01(24)	0.22(27)	-0.19(27)	-0.22(23)
	TN	0.08(23)	0.33(26)	0.06(26)	-0.16(22)
	Clay content	0.12(14)	0.4(16)	0.34(14)	-0.75(14)**
	Experimental duration	0.15(45)	-0.12(61)	0.07(59)	0.1(53)
	organic fertilizer rate	0.08(40)	-0.08(50)	0.29(48)*	0.38(44)*

MAT, mean annual temperature (°C); C input, the input rate of organic carbon; N input, the input rate of nitrogen; MAP, mean annual precipitation (mm). Soil pH, SOC and TN represent initial soil pH, organic carbon, and total nitrogen, respectively. The significant correlations are marked by asterisk (*, $P<0.05$, **, $P<0.01$, ***, $P<0.001$). The number in brackets indicates the number of observations.

Supplementary Table 2 Correlation coefficients between response of bacterial alpha diversity and climate factors, soil properties, and fertilizer regimes.

Fertilizer regime	Factor	RR of Richness	RR of Shannon
Organic fertilizer	MAT	-0.15(49)	-0.11(57)
	MAP	-0.3(49)*	-0.08(57)
	Soil pH	0.3(33)	-0.04(37)
	SOC	-0.32(36)	0.12(42)
	TN	-0.13(32)	-0.15(36)
	Clay content	-0.44(11)	-0.17(12)
	Experimental duration	-0.09(49)	0.02(57)
	organic fertilizer rate	0.28(37)	0.02(46)
Chemical-organic fertilizer	MAT	-0.09(61)	-0.04(66)
	MAP	-0.11(61)	-0.08(66)
	Soil pH	-0.03(34)	-0.01(41)
	SOC	-0.05(35)	-0.11(42)
	TN	-0.22(35)	-0.26(42)
	Clay content	-0.53(11)	-0.6(10)
	Experimental duration	-0.19(61)	-0.09(66)

MAT, mean annual temperature (°C); C input, the input rate of organic carbon; N input, the input rate of nitrogen; MAP, mean annual precipitation (mm). Soil pH, SOC and TN represent initial soil pH, organic carbon, and total nitrogen, respectively. The significant correlations are marked by asterisk (*, $P<0.05$, **, $P<0.01$, ***, $P<0.001$). The number in brackets indicates the number of observations.

Supplementary Figure

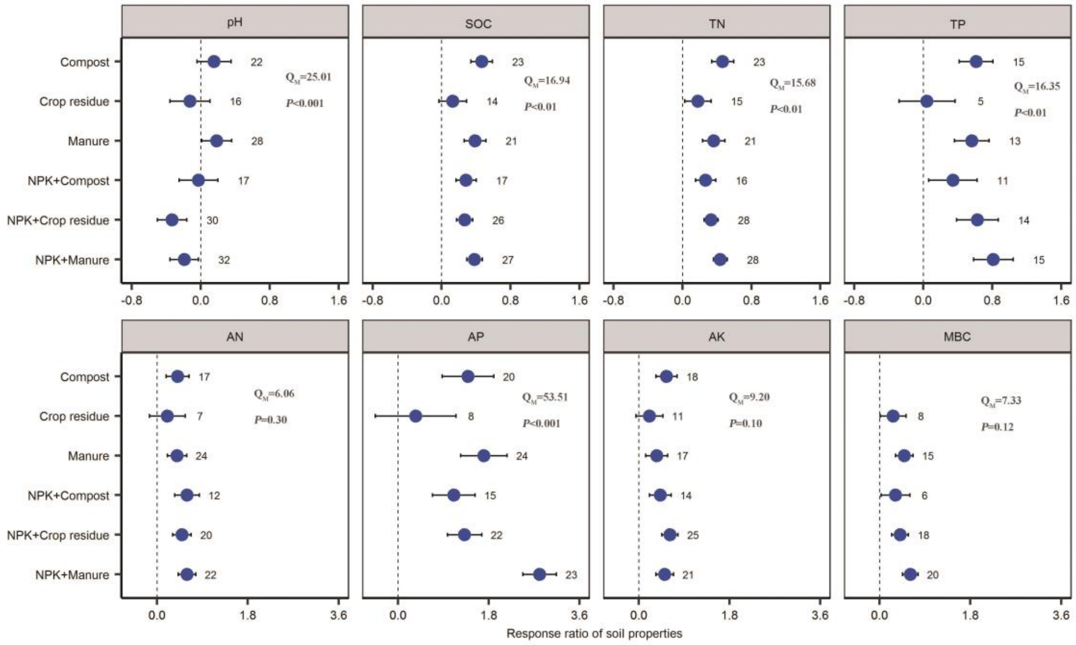

Supplementary Figure S1 Effect of different fertilizer types on soil properties. Values are mean ± 95% confidence intervals of the percentage effects between the treatment and control. The numbers at the right side of the confidence intervals represent the sample sizes. SOC, TN, TP, AN, AP, AK and MBC represent soil organic carbon, total nitrogen, total phosphorus, available nitrogen, available phosphorus, available potassium and microbial biomass carbon, respectively.

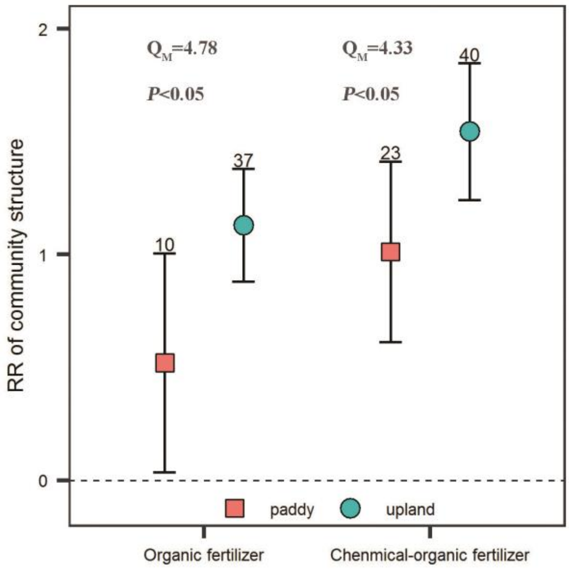

Supplementary Figure S2 Response of bacterial community structure to different land use types. Values are mean ± 95% confidence intervals of the percentage effects between the treatment and control. The numbers at the right side of the confidence intervals represent the sample sizes. OF and COF represent organic fertilizer and chemical-organic fertilizer, respectively.

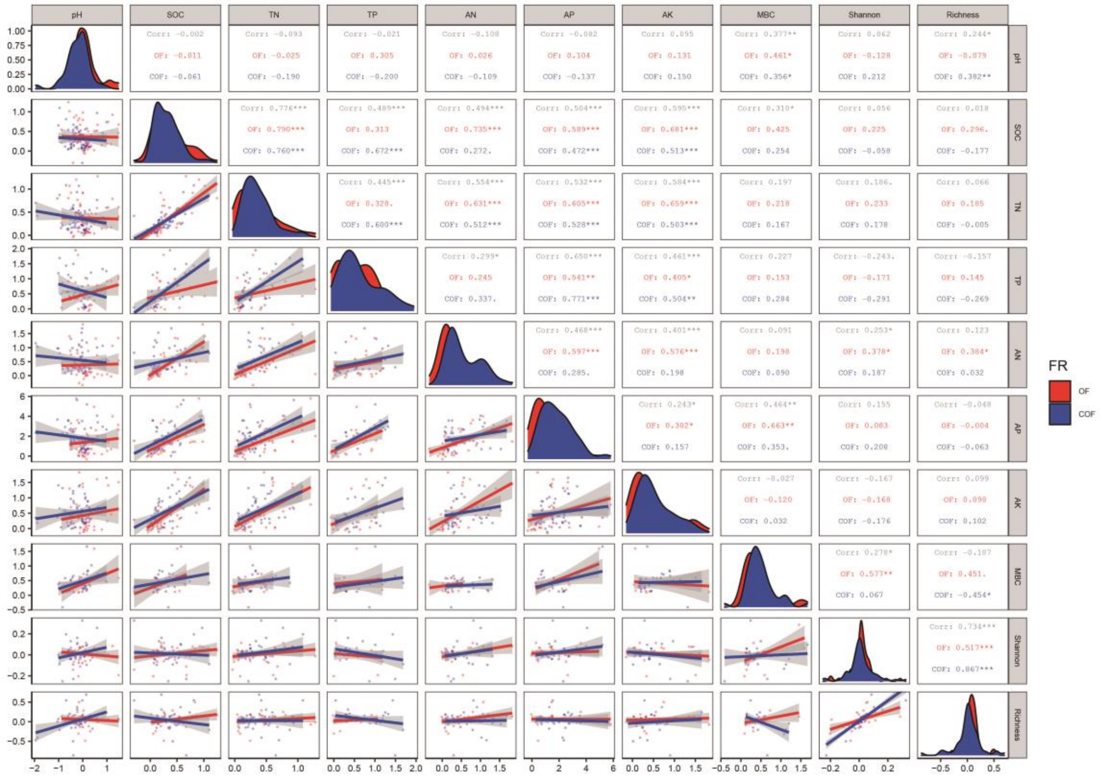

Supplementary Figure S3 Relationship between changes in soil properties and response ratio of bacterial diversity and community structure. pH, SOC, TN, TP, AN, AP, AK and MBC represent soil pH, organic carbon, total nitrogen, total phosphorus, available nitrogen, available phosphorus, and available potassium, and microbial biomass carbon respectively. OF and COF represent organic fertilizer and chemical-organic fertilizer, respectively. The significant correlations are marked by asterisk (*, *P*<0.05, **, *P*<0.01, ***, *P*<0.001).

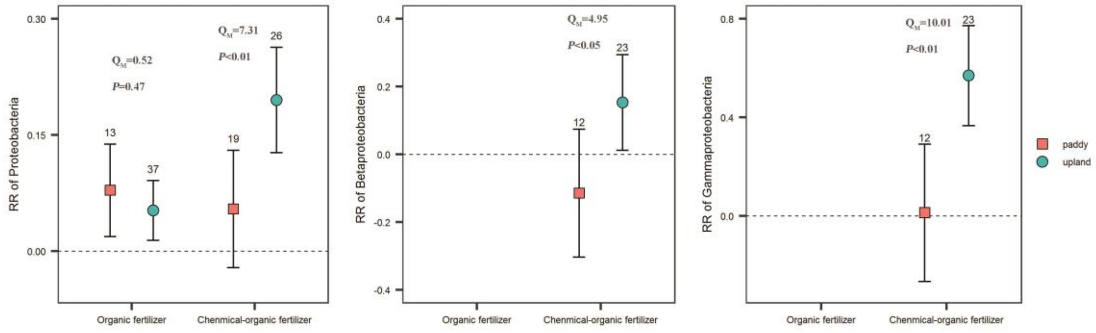

Supplementary Figure S4 Response of specific bacterial taxa to different land use types. Values are mean ± 95% confidence intervals of the percentage effects between the treatment and control. The numbers at the right side of the confidence intervals represent the sample sizes. OF and COF represent organic fertilizer and chemical-organic compound fertilizer, respectively.

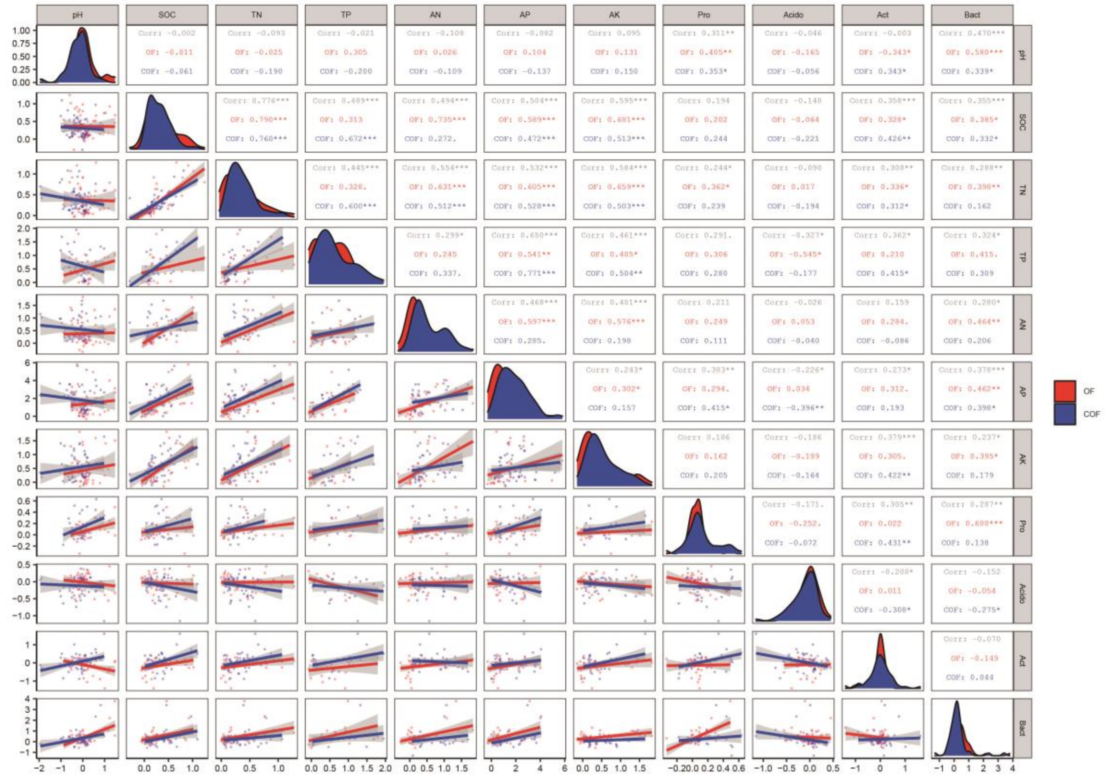

Supplementary Figure S5 Relationship between changes in soil properties and response ratio of specific bacterial phyla. pH, SOC, TN, TP, AN, AP and AK represent soil pH, organic carbon, total nitrogen, total phosphorus, available nitrogen, available phosphorus, and available potassium, respectively. Pro, Acido, Act and Bact represent Proteobacteria, Acidobacteria, Actinobacteria and Bacterodetes, respectively. OF and COF represent organic fertilizer and chemical-organic fertilizer, respectively. The significant correlations are marked by asterisk (*, P<0.05, **, P<0.01, ***, P<0.001).

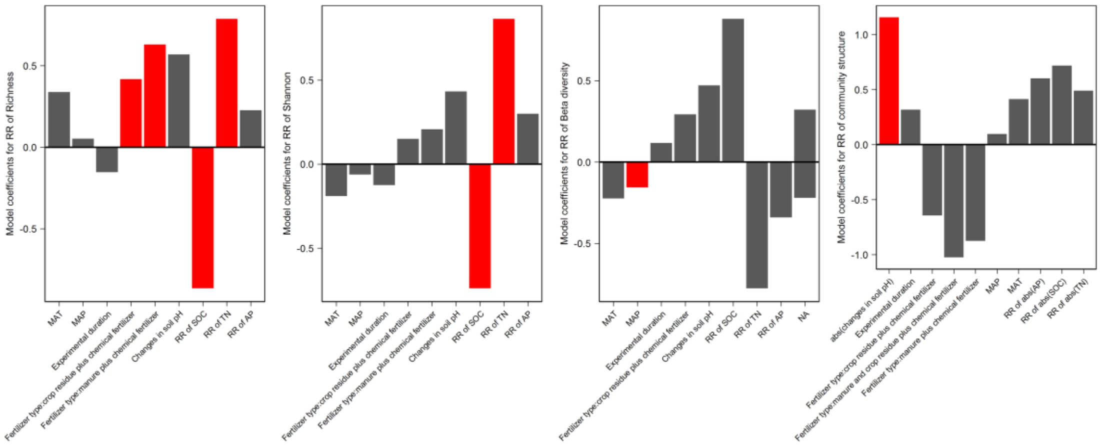

Supplementary Figure S6 Weighted averages of the model coefficients across various models for the responses of bacterial community to organic and chemical compound fertilization. The weights equal to the model probabilities. Model parameters are transferred by the fourth root for better visualization and ordered by increasing relative importance. The predictors in red bars are the ones with the sum of Akaike weights > 0.8.

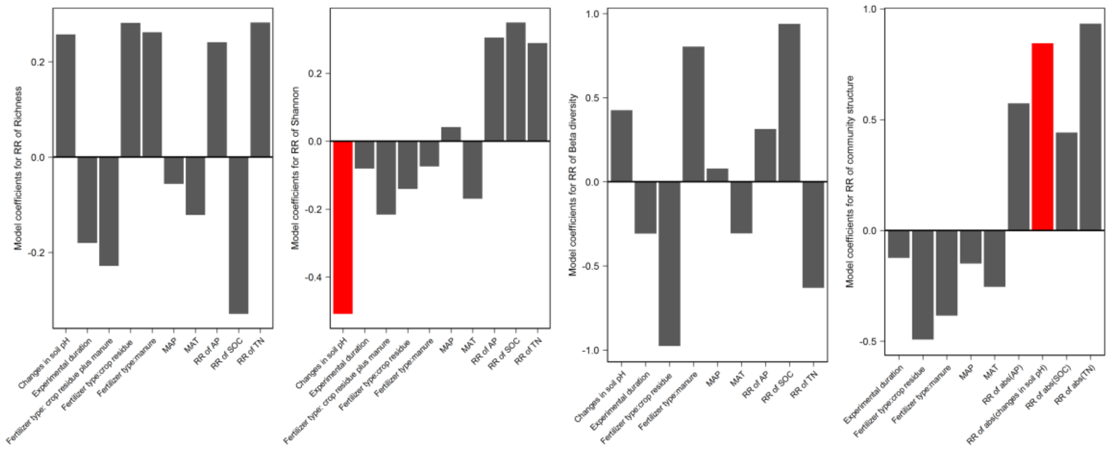

Supplementary Figure S7 Weighted averages of the model coefficients across various models for the responses of bacterial community to organic fertilization. The weights equal to the model probabilities. Model parameters are transferred by the fourth root for better visualization and ordered by increasing relative importance. The predictors in red bars are the ones with the sum of Akaike weights > 0.8.

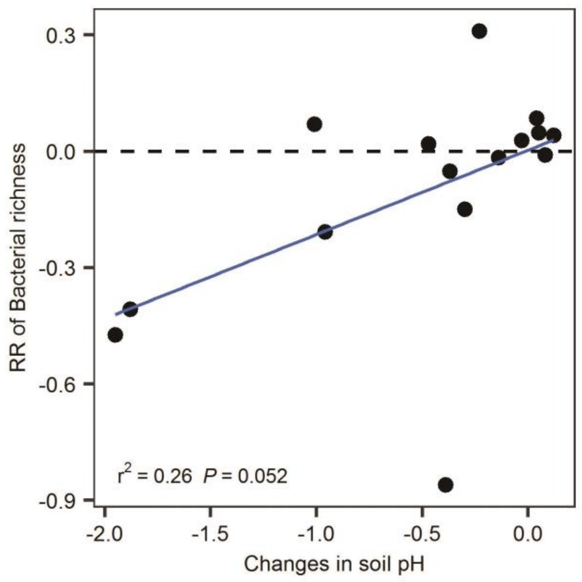

Supplementary Figure S8. Relationship between changes in soil properties and response ratio of bacterial richness under NPK+crop residue application.

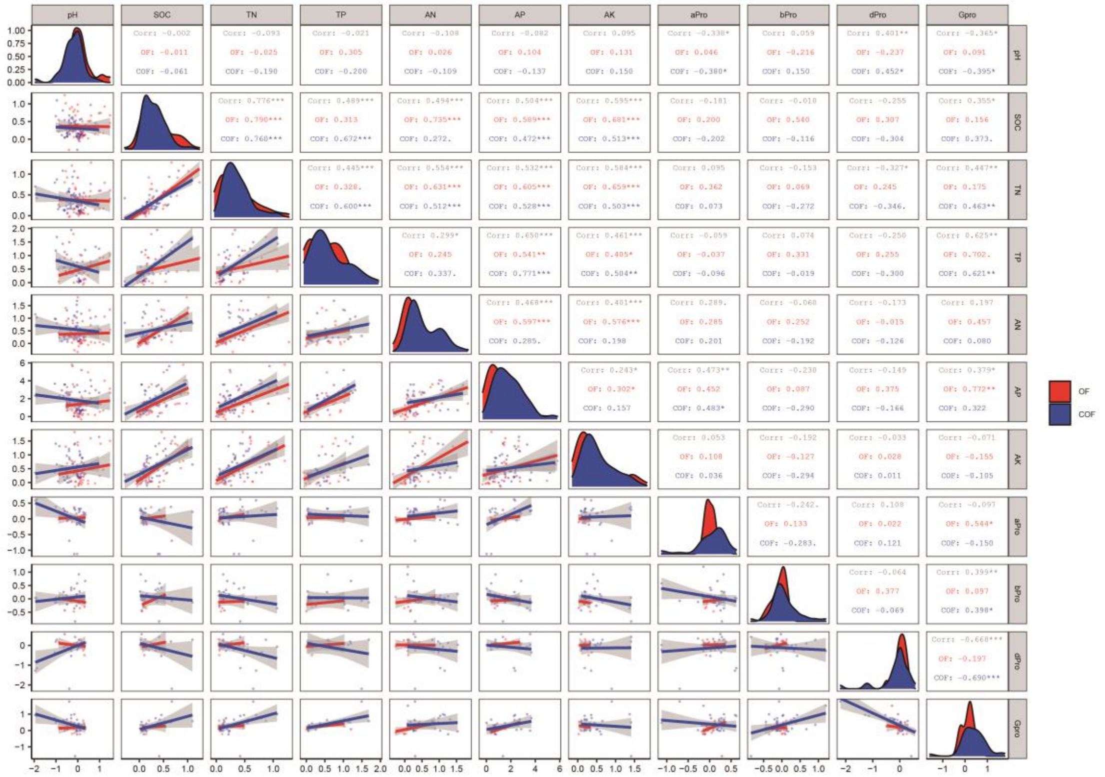

Supplementary Figure S9 Relationship between changes in soil properties and response ratio of *Proteobacterial* subclass. pH, SOC, TN, TP, AN, AP and AK represent soil pH, organic carbon, total nitrogen, total phosphorus, available nitrogen, available phosphorus, and available potassium, respectively. Alphapro,Betapro, Deltapro, and Gammapro represent *Alphaproteobacteria*, *Betaproteobacteria*, *Deltaproteobacteria*, and *Gammaproteobacteria*, respectively. OF and COF represent organic fertilizer and chemical-organic compound fertilizer, respectively. The significant correlations are marked by asterisk (*, P<0.05, **, P<0.01, ***, P<0.001).
